# Prevalence and risk factors of soil-transmitted helminthiasis among school children living in an agricultural area of North Sumatera, Indonesia

**DOI:** 10.1186/s12889-019-7397-6

**Published:** 2019-08-07

**Authors:** Ayodhia Pitaloka Pasaribu, Anggraini Alam, Krisnarta Sembiring, Syahril Pasaribu, Djatnika Setiabudi

**Affiliations:** 1 Department of Child Health, Medical Faculty, Universitas Sumatera Utara, North Sumatera, Medan, Indonesia; 2 Department of Child Health, Medical Faculty, Universitas Padjajaran, Bandung, West Java Indonesia

**Keywords:** Soil-transmitted helminth, Risk factor, School children, Indonesia

## Abstract

**Background:**

Soil-transmitted helminth infection (STH) is one of the neglected tropical disease that affects approximately 2 billion people globally. School children represent the age group that is most commonly infected with STHs, resulting in poor school performance, impaired cognitive function, and many other detrimental effects. The transmission of STH is determined by many factors, such as hygiene and sanitation. Understanding the factors that influence disease transmission in a particular area is key to effective STH control. The objective of this study was to determine the prevalence of STH in North Sumatera and to identify the associated risk factors among school children.

**Methods:**

A cross-sectional study was carried out among primary school children in Suka village, Tigapanah subdistrict. Stool samples were processed using a single Kato-Katz method. The potential risk factors analyzed were parent education and occupation, hand washing habits, latrine usage, footwear usage and contact with soil. The Chi-square test was performed to identify an association between risk factors and parasitological results. Logistic regression analysis was used to measure the strength of association.

**Results:**

We enrolled 468 school children between 6 and 12 years of age. Among those children, 268 children (57.24%) were positive for one or more STH infections. Approximately 62.39% of children played with soil/dirt every day, and only 50% regularly washed their hands after activities. Most of the children wore shoes/slippers when going outside (87.82%) and used a latrine for defecation (85.04%). Playing with soil/dirt have been shown to increase the risk of STH infections 7.53 times, while hand washing habits and latrine usage decreased the risk of STH infections 0.16 times each.

**Conclusion:**

The prevalence of STH infection in school children in Suka village, Tigapanah subdistrict is still high. Playing with soil/dirt increased the risk of infection, while hand washing habits and latrine usage decreased the risk of infection. The combined strategies of improving the personal hygiene of children and biannual deworming can reduce the risk of STH infection in school children in Suka village, Tigapanah subdistrict.

**Electronic supplementary material:**

The online version of this article (10.1186/s12889-019-7397-6) contains supplementary material, which is available to authorized users.

## Background

Infection with soil-transmitted helminths (STHs) is one of the most common neglected tropical diseases in the world. Infection with these parasites is related to poverty and, the highest prevalence occurs in low and middle-income countries where hygiene and sanitation are poor [1, 2]. It is estimated that approximately 2 billion people are infected with STHs globally [3, 4]. *Ascaris lumbricoides*, *Trichuris trichiura*, and hookworm are the most common species infecting human [5, 6]. Morbidity and mortality due to STH infections are related to the number of worms in an infected person, as well as age and immunity. School children are the most vulnerable group of people affected by the disease. In 2010, it was estimated that more than 613 million school-age children were at risk of this infection worldwide [7]. Infection of these parasites in children may result in malnutrition, poor school performance, delayed physical growth and impaired cognitive function [5, 8, 9]. The World Health Organization (WHO) is targeting the control of STH in children to reduce morbidity by 2020 through a school-based deworming program [3]. The World Health Organization developed a policy for the control of STH and schistosomiasis by promoting deworming as the cornerstone of control with the aim of reducing morbidity [10, 11]. The World Health Organization recommends annual treatment in areas where the prevalence of STH is between 20 and 50% and biannually where the prevalence is over 50% [10]. However, deworming has a temporary effect on transmission and cannot prevent reinfection [11, 12]. The transmission of STH is determined by multiple factors, such as behavior, environment, health system and socioeconomic status [13, 14]. Long-term STH control and elimination will require an integrated approach by combining deworming with the improvement of water access, sanitation (improved latrines and fecal sludge management) and hygiene (hand washing habits and wearing shoes) practices [12, 15–17]. Understanding the factors that influence an endemic area is key to effective STH control [18].

In 2010, it was estimated that 5.3 billion people, including 1 billion school children, lived in stable transmission areas for STH in the world, and nearly 70% of them lived in Asia [19]. In recent decades, Southeast Asia has been recognized as having the highest prevalence of STH infection [2]. Many countries in Southeast Asia have a moist climate that provides an ideal environment for STH embryonation and survival of eggs and larvae maturation [2, 20]. Many areas also lack adequate water resources and have poor sanitation infrastructure [2]. North Sumatera is one of the provinces in Indonesia with the highest prevalence of STH. Data reported over a decade ago, showed that the prevalence rates were between 91 and 97% in school children [21]. Since that time, some improvements have been made in the areas of water resources availability and increased use of latrines. Unfortunately, human habits have not changed much, and mass drug administration is not regularly provided (personal communication).

The objective of this study was to determine the prevalence of STH in North Sumatera and to identify the associated risk factors among school children. This is the first study to evaluate the potential risk factors of STH infection in school children in this area. The information provided could guide local policy makers to design a more definitive control strategy for STH in this area to achieve the global goal of STH control in 2020.

## Methods

### Description of study area

**Fig. 1:**
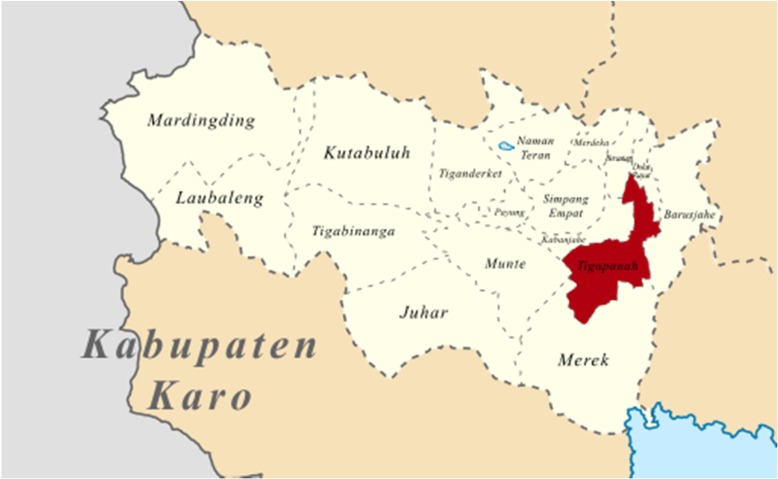
Map of Tigapanah subdistrict, Karo Regency (https://commons.wikimedia.org/wiki/Category:Tigapanah,_Karo)

The study was conducted in Suka village, Tigapanah subdistrict, Karo Regency, North Sumatera, Indonesia, from May to July 2018. Tigapanah is located 76 km from Medan, the capital city of North Sumatera. The area which covers 219,09 km^2^ is mountainous, with humid weather, wet soil and agriculture is the main occupation of its inhabitants. Figure 1 shows the study area.

### Study design and sample size

A cross-sectional study was carried out among primary school children in Suka village, Tigapanah subdistrict. This village was selected based on a previous study conducted in 2003, which reported the prevalence of STH to be 91.3% in school children [21]. The village has two adjacent elementary schools with similar characteristics and numbers of students. The sample size calculation was performed using a single proportion formula: N = P (1-p) Z^2^/d^2^, where P = prevalence of STH from a previous study, Z = level of confidence, 1.96 and d = 5% marginal error, which gave a minimum sample size of 122 children. Finally, we sought consent from parents of all invited children who attended school during sample collection and collected stool samples the next day for examination. Sample collection until data analysis was conducted from May to July 2018. Each of the children was provided with a unique identification (ID) code to identify each individual and avoiding repetitive sampling.

### Data collection

Characteristic data, such as age, sex, nutritional status, and parent occupation and education, were collected. Children were interviewed about their self-hygiene, such as hand washing before and after defecation, latrine and footwear usage and whether they enjoyed playing with soil/dirt. That information was collected using a pretested questionnaire that was prepared for the study. It was prepared in Bahasa and checked for fitness (English translated version is provided as Additional file 1). Trained study team members conducted the interview. All questionnaires were checked for completeness and accuracy.

The heights of all of the children were examined using a calibrated ruler to the nearest 0.1 cm. Weight was measured using a calibrated scale to the nearest 0.5 kg without shoes. Height for age (HAZ) and weight for age (WAZ) scores were used to determine growth rate and nutritional status.

### Stool sample examination

The children were provided with plastic containers that were already labeled with their unique ID and date and kept in a plastic bag. They were instructed to bring the stool sample the next morning. Diarrheic sample was withdrawn to maintain the quality of the stool samples. The samples were checked for quality and then emulsified with 10% formalin solution before being transported to Universitas Sumatera Utara in Medan for examination. Stool samples were processed using a single Kato-Katz smear and microscopically examined for ova of intestinal helminths. The intensity of infection was determined as the eggs per gram (epg) of stool according to WHO guidelines [22].

### Data analysis

Data were analyzed using STATA version 15.1 (Stata Corporation, College Station, Texas, USA) after checking for completeness. Descriptive data were summarized as a percentage and interquartile ranges (IQR). The potential risk factors analyzed were parent education and occupation, hand washing habits, latrine usage, footwear usage and contact with soil. Logistic regression analysis was used to explain the relationship between independent variables and STH infection. Odds ratio (OR) of the binary outcome variable was assessed by univariate analysis. All variables with *P*-value less than 0.25 were further analyzed using multivariate analysis to adjust any confounders. The final model was presented by adjusted odds ratio (AOR) with 95% confidence interval (CI). Values were considered significant when *P* < 0.05.

### Ethical consideration

This study was approved by the Health Research Ethical Committee, Medical Faculty of Sumatera Utara (No. 233/TGL/KEPK FK USU-RSUP HAM/2018) and Research Ethics Committee, Universitas Padjajaran (No. 650/UN6.KEP/EC/2018). Permission to conduct the study was also approved by the Tigapanah subdistrict office, educational authorities and school principals.

The study objective was explained to the parents, school teachers, and students before data collection. Written consent was obtained from parents before data and stool collection. Any children with positive STH infection from stool examination were treated with an appropriate dose of albendazole (chewing tablet of albendazole, 400 mg) provided by the Ministry of Health.

## Results

### Characteristics of the study population

From the descriptive analysis, we found that 51.07% of school children were boys, and the mean age of children included in this study was 9.4 (standard deviation of 4.22) years. The occupation of the majority of parents was farming both for mothers and fathers (91.67 and 79.7%, respectively). Approximately 62.39% of children played with soil/dirt every day, and only 50% regularly washed their hands after activities. Most of the children wore shoes/slippers when going outside (87.82%) and used a latrine for defecation (85.04%). Detailed information about the characteristics of these children is provided in Table 1.

**Table 1:** Characteristics of school children enrolled in the study

Characteristics observed	School children (*N* = 468, %)
Sex	
Girl	229 (48.93)
Boy	239 (51.07)
Age group	
6–10 years old	295 (63.03)
Above 10 years old	173 (36.97)
Mother occupation	
Farming	429 (91.67)
Others	39 (8.33)
Father occupation	
Farming	373 (79.70)
Others	95 (20.30)
Mother education	
No education	68 (14.53)
Primary	120 (25.64)
Secondary	246 (52.56)
High school	34 (7.26)
Father education	
No education	101 (21.58)
Primary	156 (33.33)
Secondary	195 (41.67)
High school	16 (3.42)
Nutritional status	
Average	315 (69.31)
Underweight	93 (19.87)
Overweight	60 (12.82)
Playing with soil/dirt	292 (62.39)
Wearing shoes/slippers	411 (87.82)
Latrine usage	398 (85.04)
Hand washing habit	234 (50)

### Prevalence of soil-transmitted helminthiasis

Between May and July 2018, we screened 510 school children from 2 elementary schools in Suka village, Tigapanah subdistrict. Four of the children did not participate because their parents did not provide consent, and 38 children could not be included because they did not provide stool samples. We enrolled 468 school children between 6 and 12 years of age. Among those children, 268 children (57.24%) were positive for one or more STH infections. We also found 2 children infected with *Hymenolepis nana* and 1 with *Enterobius vermicularis*.

The most frequently identified species of STH was *A. lumbricoides* (40.17%) (Table 2).

**Table 2:** Prevalence and multiplicity of soil-transmitted helminth infection in school children in Suka village

Variables	Number positive (N = 468)	Prevalence (%)
STH Species		
*Ascaris lumbricoides*	188	40.17
*Trichuris trichiura*	156	33.33
Hookworms	16	3.42
Infection types		
Mono-infection	182	67.91
Dual infection	83	30.97
Triple infection	3	1.12
Gender		
Female	133	49.6
Male	135	50.4
Age group		
6–10 years	170	63.4
> 10 years	98	36.6

We found 182 children with single infection of STH (67.91%), 83 children with double STH infections (30.97%) and three children with triple STH infections (1.12%).

In this study, we only detected light-intensity infections for all species of STH, based on egg per gram. For *A. lumbricoides*, the geometric mean epg was 984.34 (IQR: 766.31–1264.40), followed by *T. trichiura* with 108.24 epg (IQR: 91.42–128.15) and hookworms with 113.90 epg (IQR: 66.93–193.83).

### Potential risk factors for soil-transmitted helminth infection in school children

The result of the logistic regression analysis is shown in Table 3. Playing with soil/dirt increased the risk of STH infection 7.53 times while each of hand washing habits and latrine usage decreased the risk by 0.16.

**Table 3:** Factors associated with soil-transmitted helminth infection in school children in Suka village

Risk factors	Total examined N = 468		Bivariate analysis		Multivariate analysis		
	STH (*n* = 268)	No STH (*n* = 200)	OR	95% CI	AOR	95% CI	*P*-value
Mother occupation			0.77	0.39–1.48			
Farming	248	181					
Others	20	19					
Father occupation			1.03	0.66–1.63			
Farming	213	160					
Others	55	40					
Mother education							
No education	40	28					
Primary	74	46	1.13	0.61–2.07			
Secondary	139	107	0.91	0.53–1.57			
High school	15	19	0.55	0.24–1.27			
Father education							
No education	64	37					
Primary	99	57	1.004	0.59–1.69	1.11	0.60–2.07	
Secondary	98	97	0.58	0.36–0.96	0.69	0.38–1.23	
High school	7	9	0.45	0.16–1.31	0.61	0.17–2.15	
Playing with soil/dirt	126	166	1.87	1.61–2.17	7.53	4.54–12.49	< 0.001
Not wearing shoes/slippers	47	10	4.041	1.99–8.21	1.82	0.67–4.93	
Latrine usage	59	11	0.21	0.10–0.40	0.16	0.07–0.35	< 0.001
Hand washing habit	177	57	0.21	0.14–0.30	0.16	0.10–0.26	< 0.001

We also analyzed the association between potential risk factors and STH species. For infection with *A. lumbricoides*, playing with soil/dirt increased the risk of infection 2.56 times while hand washing habits and latrine usage decreased the risk of infection by 0.38 and 0.30 times, respectively. For infection with *T. trichiura*, playing with soil/dirt increased the risk of infection by 4.44 times, while hand washing habits decreased the infection 0.31 times. We did not perform a logistic regression analysis for hookworm because the sample size was too small.

## Discussion

Our study showed that soil-transmitted helminth infections are prevalent among school children in Suka village, Tigapanah subdistrict. The present finding shows that the prevalence of STH decreased by over 34% when compared with the report of Pasaribu [21]. This finding suggests improvement in water resources and sanitation infrastructure in the area. The finding is however higher than the reports of 7.6 and 34.4% in Northern Sumatera [23] and Suka village [24] respectively. We enrolled more children less than ten years old in this study, which was similar to the study by Alemeshet [25].

In the present study, *A. lumbricoides* was the most predominant STH species, followed by *T. trichiura* (33.33%) and then hookworms (3.42%) This finding is similar to the report of Wang et al. [26] in China, but contrary to the reports of Alelign et al. [27] in North-western Ethiopia which reported predominance of hookworms. Predominance of *T. trichiura* over other species of STH was also reported in Ethiopia and Cote d’Ivore [13, 28]. It was estimated that *A. lumbricoides* is attributed to the highest burden of STH infection in the world and is most commonly found in children, while hookworms are more common in adults. Approximately 87.82% of the children in our study wore shoes while playing outside, which contributed to the reduced number of hookworm infections. Interestingly, we also found some children in our study who were infected with *Hymenolepis nana* and *Enterobius vermicularis*. We did not find any infection with *S. stercoralis*, probably because of the use of Kato Katz method, which is limited in detecting the parasite [29].

In line with the report of Abossie et al. [30] from southern Ethiopia, we found mono, dual and triple infections in 67.91, 30.97 and 1.12% of children respectively. This finding is however contrary to other reports from Ethiopia [25, 30, 31]. Most of the dual infections observed were due to *A. lumbricoides* and *T. trichiura* similar to the report of Debalke et al. [28]. The global predominance of these two species and the humid weather and wet soil in Suka village may explain their abundance in the study region. The prevalence of triple infections was lower than reports from Honduras [14, 32]. Multi-parasitism were also reported in Asia [33, 34].

A study by Erismann in Burkina Faso showed moderate-intensity of STH infections [35], while in our study all infections were of light intensity. However, our result is similar to the finding of Davis et al. [36] which also showed light- intensities of STH infections. Although the number of epg was considered low, STH infection may have a negative impact on children’s health and quality of life, as shown by Al-Mekhlafi in Yemen [37].

Our study assessed the association of potential risk factors with the prevalence of STH infection. Parents education and occupation, the habit of playing with soil/dirt, not wearing shoes/slippers, latrine usage, and hand washing habits were the factors that we used in the analysis. We did not find any association between parent education and occupation with the risk of STH infection. A related study by Terefa [38] also showed similar result. However Wang [26], showed that mother’s education level contributed to the occurrence of STH infection contrary to the present finding. Playing with soil/dirt increased the risk of STH 7.53 times, while latrine usage and hand washing habit significantly reduced the risk of STH by 0.16 times each. Studies carried out in Honduras and Malaysia also confirmed the fact that open defecation can increase the risk of polyparasitism by nearly two times [14, 39, 40]. From multivariate analysis, we did not find an association between wearing shoes/slippers and the risk of STH infection. This finding is similar to those in the study performed by Ahmed in Malaysia [41].

When we analyzed the association between species of STH and the prevalence of infection, playing with soil/dirt increased the risk for both *A. lumbricoides* 2.56 times and *T. trichiura* infections 4.44 times. Hand washing habits decreased the risk of both *A. lumbricoides* and *T. trichiura* infections, 0.38 and 0.31 times, respectively. Latrine usage decreased the risk of *A. lumbricoides* infection 0.30 times but had no association for *T. trichiura* infection.

A study conducted by Tabi et al. [42] also showed that open defecation is a risk factor for STH infections. Lack of hand washing was also reported as a risk factor for STH infection [43].

This is the first study to evaluate the risk factors of personal hygiene in school children and their associations with STH infection in Suka village. Our study findings will inform public health authorities to develop a relevant STH control strategy in Suka village, Tigapanah subdistrict.

## Conclusions

The prevalence of STH infection in school children in Suka village, Tigapanah subdistrict is still high. Children’s personal hygiene was associated with the prevalence of STH, but we found no association between parent’s education and occupation with the prevalence of STH. Playing with soil/dirt increased the risk of infection, but hand washing habits and latrine usage potentially decreased the risk of infection. The combined strategies of improving the personal hygiene of children and biannual deworming will reduce the risk of STH infection in school children in Suka village, Tigapanah subdistrict.

## Additional file

Additional file 1: English translated version of study questionnaire (Interview guide). This file is an English translated version of study questionnaire. The original version is in Bahasa Indonesia and was used in participant interview. (DOCX 19 kb)

## Data Availability

The data sets in this study are available from the corresponding author on reasonable request.

## Abbreviations

A. lumbricoides: Ascaris lumbricoides
AOR: Adjusted odds ratio
CI: Confidence interval
EPG: Egg per gram
HAZ: Height for age
ID: Identification code
IQR: Interquartile range
OR: Odds ratio
STH: Soil transmitted-helminth
T. trichiura: Trichuris trichiura
WAZ: Weight for age
WHO: World Health Organization
