# Supplementary material for: Prevalence and risk factors of soil-transmitted helminthiasis among school children living in an agricultural area of North Sumatera, Indonesia
Source: BMC Public Health. 2019 Aug 7;19:1066. doi: 10.1186/s12889-019-7397-6 (PMC6686497; doi:10.1186/s12889-019-7397-6)
Supplement: Supplementary file 1 — English translated version of study questionnaire (Interview guide). This file is an English translated version of study questionnaire. The original version is in Bahasa Indonesia and was used in participant interview. (DOCX 19 kb) [file 12889_2019_7397_MOESM1_ESM.docx]

**Risk Factor of STH among school children**

Research Questionnaire

Participant ID: __ __ __

Date/time: __ __ __ __ __ __ / __ __ :__ __

1. **Personal Identity**
2. Gender: Male / Female
3. Date of birth/Age: __ __ __ __ __ __ / __ __
4. Body weight: __ __kg
5. Body Height: __ __cm
6. History of antihelminthic consumption within 1 month? Yes/No
7. **Parents Information**
8. Mother Education:
9. No Education
10. Elementary school
11. Secondary school
12. High School
13. Mother occupation:
14. Farming
15. Others, specify : ______________________
16. Father Education:
17. No Education
18. Elementary school
19. Secondary school
20. High School
21. Father occupation:
22. Farming
23. Others, specify : ______________________
24. **Personal Hygiene**
25. Do you have latrine at home?
26. Yes (1) b. No (0)
27. Do you usually use latrine to defecate?

a. Yes (1) b. No (0)

1. Do you have latrine at school?

a. Yes (1) b. No (0)

1. Do you wash your hands after going to toilet?

a. Yes (1) b. No (0)

1. Do you wash your hand before eating?

a. Yes (1) b. No (0)

1. Do you wash your hand after playing?

a. Yes (1) b. No (0)

1. Do you wear shoes/slippers when you play in the garden?

a. Yes (1) b. No (0)

1. Do you wear shoes/slippers at school?

a. Yes (1) b. No (0)

1. Do you wear shoes/slippers at home?

a. Yes (1) b. No (0)

1. Do you usually play with soil outside your house?

a. Yes (1) b. No (0)

1. Do you usually play with soil at school?

a. Yes (1) b. No (0)

1. Do you usually play with soil in the garden?

a. Yes (1) b. No (0)

Interview done by : _______________________

Signature : ______________________________
